# Supplementary material for: Acute and endothelial-specific Robo4 deletion affect hematopoietic stem cell trafficking independent of VCAM1
Source: PLoS One. 2021 Aug 13;16(8):e0255606. doi: 10.1371/journal.pone.0255606 (PMC8362960; doi:10.1371/journal.pone.0255606)
Supplement: S1 Fig — A. Robo4 germline knockout model [18,40]. Alkaline phosphatase was inserted between exons 2–4. B. Schematic of tamoxifen injection schedule. Mice (Figs 4–6) were analyzed on day 7, 3 days after final IP injection of tamoxifen. C. Robo4 conditional knockout model [19], RosaCreERT2 [20], and VeCadherin-CreERT2 [21]. LoxP sites were inserted flanking exon 3 of the Robo4 gene. D. Genetic deletion in the Robo4 locus and reduced Robo4 mRNA levels in the three mouse models. Di. Image depicted shows PCR-based banding patterns for DNA isolated from ear clips of R4lox/lox, Wt, and R4cKO (in duplicate) mice, VECs sorted from Wt tamoxifen-treated mice (in triplicate), R4cKO tamoxifen-treated mice (in triplicate), and R4ECcKO tamoxifen-treated mice (in triplicate). The estimated banding patterns: untreated R4lox/lox: 903bp, Wt: 601 bp, and tamoxifen-treated R4cKO and R4ECcKO (floxed exon deleted): 400bp. (Relative gene expression of mRobo4. (ii) The VEC and (iii) HSC populations were isolated by FACS from untreated (no tamoxifen) Wt or R4KO mice or from Wt, R4cKO, and R4ECcKO mice post tamoxifen treatment (treatment schedule as in panel C). Robo4 mRNA levels were normalized to β-actin levels. Robo4 mRNA levels were reduced in VECs in all 3 models, as expected (panel Dii). In HSCs, Robo4 mRNA levels were reduced in R4KO and R4cKO, but not in R4ECcKO, mice, also as expected (panel Diii). Data represent n = 3 experiments from 2–3 mice per cohort per sort. Statistics by unpaired two tailed student’s t-test ** p < 0.01 and **** p < 0.0001. (PDF) [file pone.0255606.s001.pdf]

Figure S1

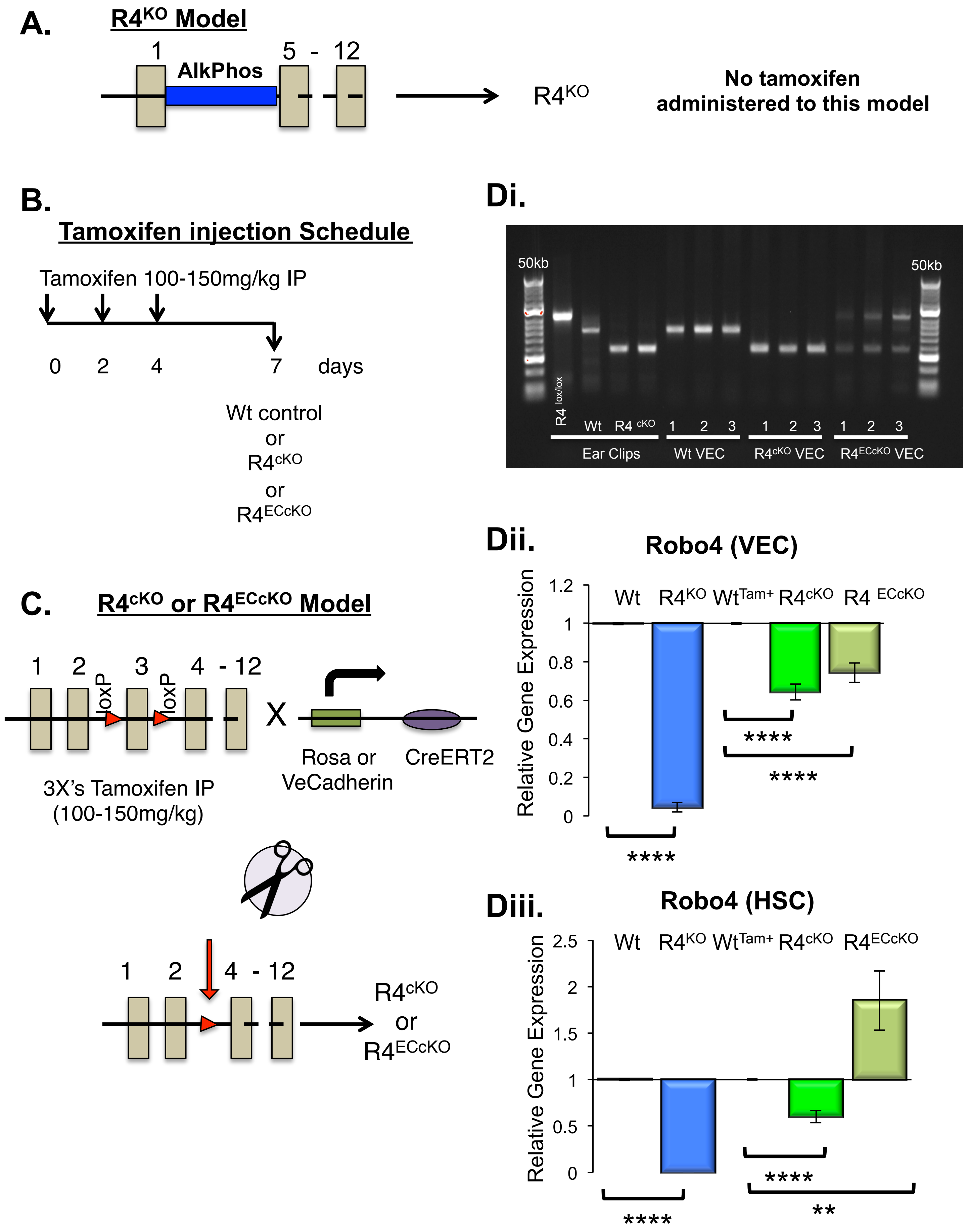
A.
R4KO Model
1
AlkPhos
5 - 12
R4KO
No tamoxifen administered to this model
B.
Tamoxifen injection Schedule
Tamoxifen 100-150mg/kg IP
0 2 4 7 days
Wt control or R4cKO or R4ECcKO
C.
R4cKO or R4ECcKO Model
1 2 3 4 - 12
loxP
loxP
X
Rosa or VeCadherin
CreERT2
3X's Tamoxifen IP (100-150mg/kg)
1 2 4 - 12
R4cKO or R4ECcKO
Di.
50kb
50kb
R4 lox/lox
Wt R4 cKO 1 2 3 1 2 3 1 2 3
Ear Clips
Wt VEC
R4cKO VEC
R4ECcKO VEC
Dii.
Robo4 (VEC)
Relative Gene Expression
1.2
1
0.8
0.6
0.4
0.2
0
Wt R4KO WtTam+ R4cKO R4 ECcKO
****
****
****
Diii.
Robo4 (HSC)
Relative Gene Expression
2.5
2
1.5
1
0.5
0
Wt R4KO WtTam+ R4cKO R4ECcKO
****
****
**
