# Supplementary figures and images for: Acute and endothelial-specific Robo4 deletion affect hematopoietic stem cell trafficking independent of VCAM1
Source: PLoS One. 2021 Aug 13;16(8):e0255606. doi: 10.1371/journal.pone.0255606 (PMC8362960; doi:10.1371/journal.pone.0255606)

**Figure S2**

**A.**

**MSC**

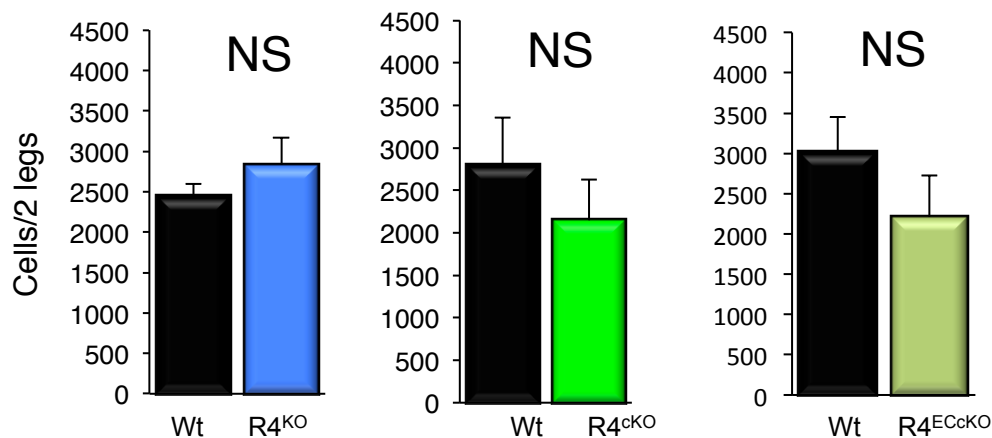

**B.**

**OBL**

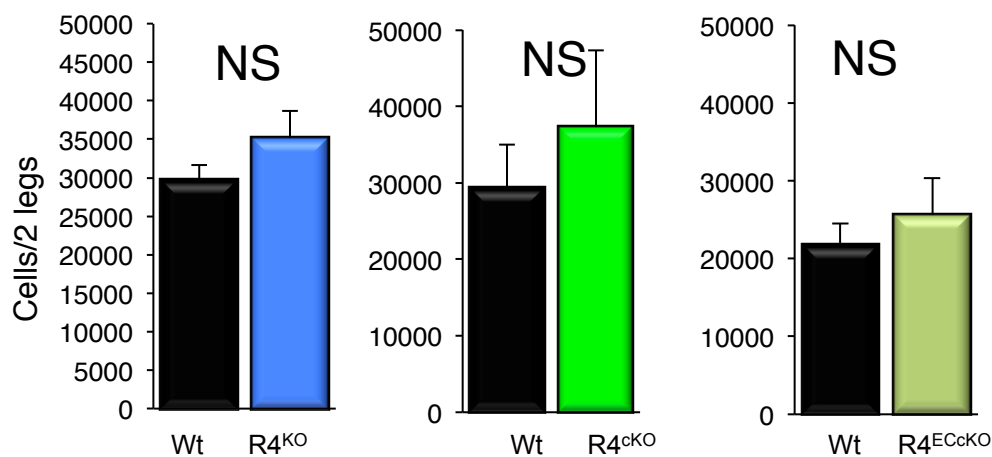

**C.**

**CD45<sup>+</sup>**

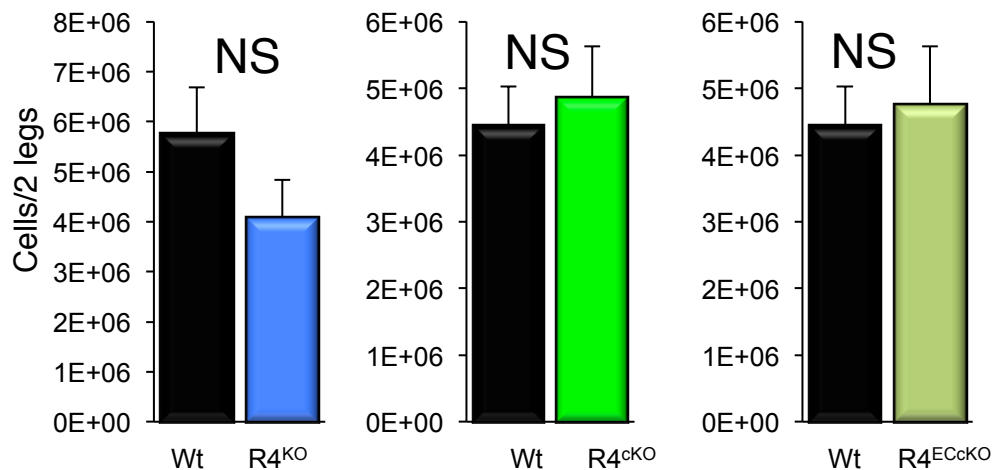

Supplement: S2 Fig — A. Germline, acute, or endothelial-specific deletion of Robo4 did not result in significantly altered cell numbers of bone marrow mesenchymal stem cells (MSCs). Data represent n = 4–6 independent experiments with n = 37 Wt, n = 37 R4KO, n = 18 tamoxifen treated Wt, n = 15 R4cKO, and n = 10 R4ECcKO. Statistics by unpaired two tailed student’s t-test showing no significance. B. Germline, acute, or endothelial-specific deletion of Robo4 did not result in significantly altered cell numbers of bone marrow osteoblasts (OBL). Data represent n = 4–6 independent experiments with n = 37 Wt, n = 37 R4KO, n = 18 tamoxifen treated Wt, n = 15 R4cKO, and n = 10 R4ECcKO. Statistics by unpaired two tailed student’s t-test, showing no significance. C. Germline, acute, or endothelial-specific deletion of Robo4 did not result in significantly altered cell numbers of bone marrow CD45+ hematopoietic cells. Data represent n = 4–6 independent experiments with n = 10 Wt, n = 10 R4KO, n = 12 tamoxifen treated Wt, n = 12 R4cKO, and n = 14 R4ECcKO. Statistics by unpaired two tailed student’s t-test, showing no significance. (PDF) [file pone.0255606.s002.pdf]

Figure S3

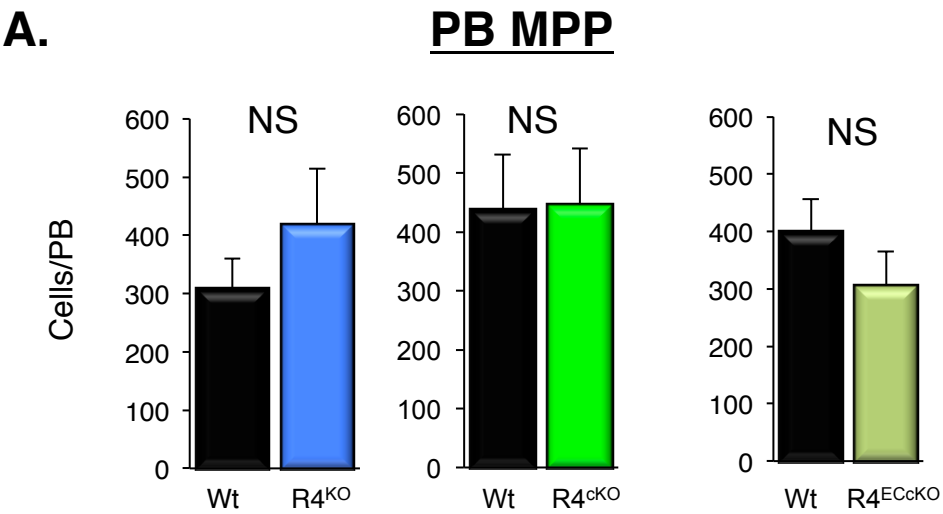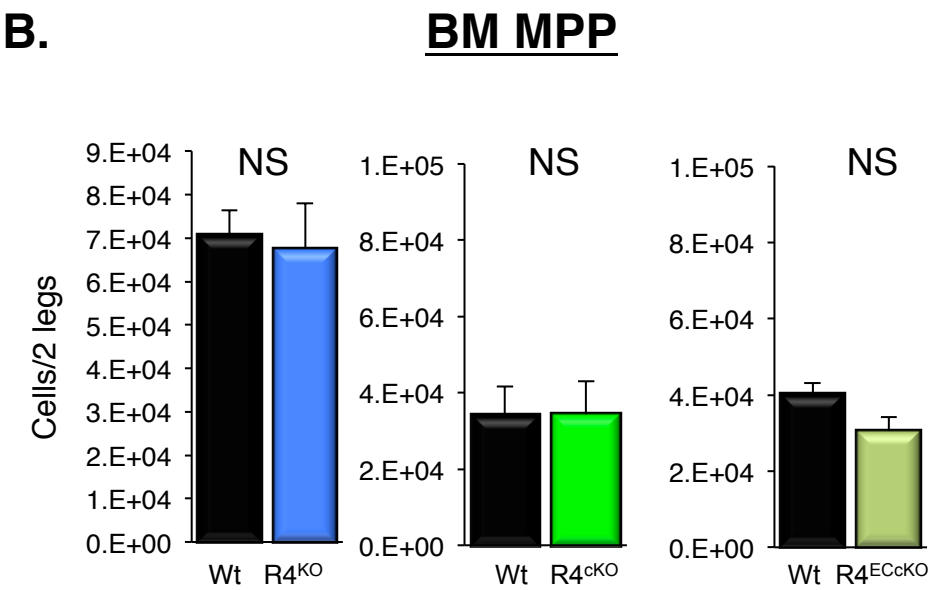

Supplement: S3 Fig — A. Unlike HSCs, MPPs were not significantly increased in the blood of Robo4-deficient mice. We have previously published similar results for the R4KO mice; this panel includes both previously reported and new data from the R4KO performed side-by-side with the two conditional models [16]. Data represent n = 5–9 independent experiments, with n = Wt, n = R4KO, n = 33 Tamoxifen treated Wt, n = 29 R4cKO, and n = 18 R4ECcKO mice. Statistics by unpaired two tailed student’s t-test, showing no significance. B. Unlike HSCs, MPPs were not significantly decreased in the BM of Robo4-deficient mice. We have previously published similar results for the R4KO mice; this panel includes previously reported data from the R4KO performed side-by-side with the two conditional models [16]. Data represent n = 5–9 independent experiments, with n = 25 Wt, n = 9 R4KO, n = 12 Tamoxifen treated Wt, n = 16 R4cKO, and n = 10 R4ECcKO mice. Statistics by unpaired two tailed student’s t-test, showing no significance. (PDF) [file pone.0255606.s003.pdf]

Figure S4

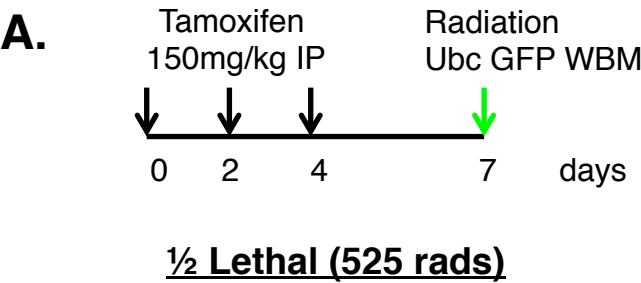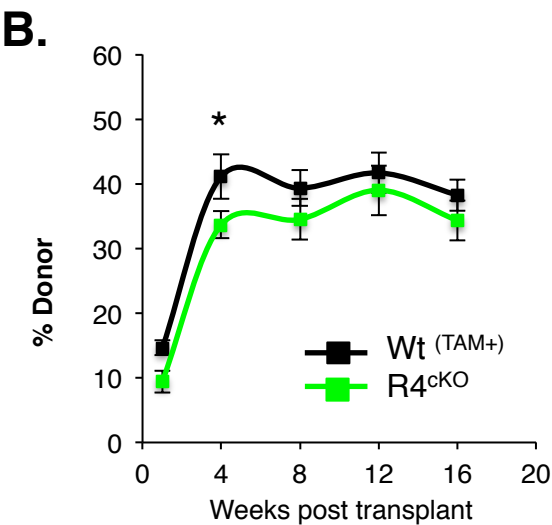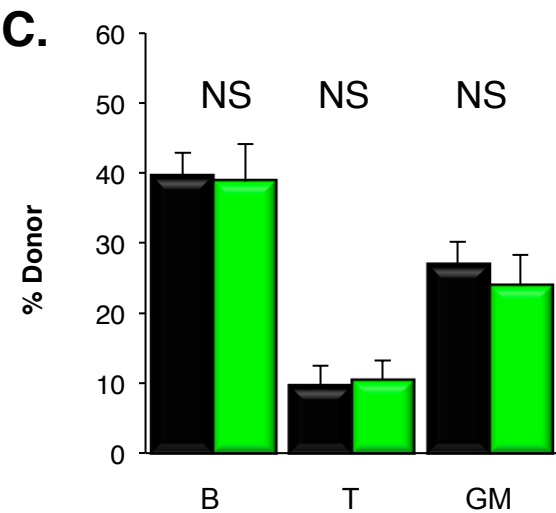

Supplement: S4 Fig — A. Schematic of tamoxifen injection schedule and radiation/transplantation 3 days post final tamoxifen injection. B. Equivalent peripheral blood donor chimerism levels in R4cKO hosts compared to Wt hosts treated as in A. Statistics by unpaired two tailed student’s t-test, showing no significance. C. No difference in donor lineages in the transplanted mice from B between R4cKO or controls. Recipients were transplanted with 7.5 M UbcGFP+ WBM cells (500 HSC equivalent). n = 3 independent experiments per radiation dose with n = 8 tamoxifen treated Wt and n = 11 R4cKO. Statistics by unpaired two tailed student’s t-test, showing no significance. (PDF) [file pone.0255606.s004.pdf]
